# Integrated Transcriptomics and Metabolomics Analysis Reveal Key Metabolism Pathways Contributing to Cold Tolerance in Peanut

**DOI:** 10.3389/fpls.2021.752474

**Published:** 2021-11-24

**Authors:** Xin Wang, Yue Liu, Zhongkui Han, Yuning Chen, Dongxin Huai, Yanping Kang, Zhihui Wang, Liying Yan, Huifang Jiang, Yong Lei, Boshou Liao

**Affiliations:** Key Laboratory of Biology and Genetic Improvement of Oil Crops, Ministry of Agriculture and Rural Affairs, Oil Crops Research Institute of the Chinese Academy of Agricultural Sciences, Wuhan, China

**Keywords:** *Arachis hypogaea*, RNA-Seq, metabolome, carbohydrate, polyamine, lignin, phenylpropanoid

## Abstract

Low temperature (non-freezing) is one of the major limiting factors in peanut (*Arachis hypogaea L.*) growth, yield, and geographic distribution. Due to the complexity of cold-resistance trait in peanut, the molecular mechanism of cold tolerance and related gene networks were largely unknown. In this study, metabolomic analysis of two peanut cultivars subjected to chilling stress obtained a set of cold-responsive metabolites, including several carbohydrates and polyamines. These substances showed a higher accumulation pattern in cold-tolerant variety SLH than cold-susceptible variety ZH12 under cold stress, indicating their importance in protecting peanut from chilling injuries. In addition, 3,620 cold tolerance genes (CTGs) were identified by transcriptome sequencing, and the CTGs were most significantly enriched in the “phenylpropanoid biosynthesis” pathway. Two vital modules and several novel hub genes were obtained by weighted gene co-expression network analysis (WGCNA). Several key genes involved in soluble sugar, polyamine, and G-lignin biosynthetic pathways were substantially higher and/or responded more quickly in SLH (cold tolerant) than ZH12 (cold susceptible) under low temperature, suggesting they might be crucial contributors during the adaptation of peanut to low temperature. These findings will not only provide valuable resources for study of cold resistance in peanut but also lay a foundation for genetic modification of cold regulators to enhance stress tolerance in crops.

## Introduction

Peanut (*Arachis hypogaea* L.) is an important oil and economic crop worldwide, and provides a rich source of nutrients for humans, including fat, protein, sugar, fatty acids, and free amino acids (Zhao et al., 2012; Sorita et al., 2020). Low temperature is one of the major environmental stresses that adversely affect plant development and geographic distribution (Kakani et al., 2002; Wang et al., 2003). The growth of peanut is severely inhibited below 15°C, and chilling stress (non-freezing) is deemed as a limiting factor in peanut cultivation and production (Bell et al., 1994). In northeast China, cold weather often occurs during spring sowing and autumn harvest seasons, leading to a serious decrease of peanut yields per year (Chen et al., 2014). With the increasing demand for peanut in recent years, it is urgent to investigate the mechanism of cold tolerance and cultivate new cold-resistant varieties.

Cold stress causes excessive accumulation of reactive oxygen species (ROS) during respiration and photosynthesis processes in plant, thus increasing cell membrane permeability and the lipid peroxidation level (Zhang et al., 2019). Nevertheless, plants evolve a series of sophisticated mechanisms to defense against cold stress to some extent (Ding et al., 2020). The alterations of gene expression in response to low temperature are a common strategy to cope with cold damage in plants (Suzuki, 2019). During the last two decades, a number of components involved in the cold-responsive-signaling pathway have been isolated and characterized, including messenger molecules (e.g., Ca^2+^), Ca^2+^-related protein kinases and several crucial transcription factors (Yuan et al., 2018). One of the best studied examples was the plant ICE-CBF-COR-signaling module (Chinnusamy et al., 2003). C-repeat Binding Factors/Dehydration-Responsive Element-Binding proteins (CBFs/DREBs) were members of APETALA 2/Ethylene Response Factor (AP2/ERF) family and played central roles in cold acclimation (Kim et al., 2015). The transcript levels of CBFs were sharply upregulated by Inducer of CBF Expression protein (ICE), an MYC-type basic helix-loop-helix family transcription factor, and then CBFs activated the expression of downstream cold-responsive (COR) genes *via* binding to *cis*-elements in their promoters (Lee et al., 2005; Tang et al., 2020). In addition, a lot of cold-resistant proteins (e.g., late embryogenesis-abundant proteins, IEAs) and protective substances (e.g., soluble sugars and proline) were synthesized in plant cells, functioning as osmolytes to regulate osmotic potential and maintain membrane integrity (Dong et al., 2002; Zuther et al., 2004; Ma et al., 2009).

Transcriptomic analysis has been a highly efficient way to reveal cold-responsive genes in many crops, such as maize (Li et al., 2019), rice (Pan et al., 2020), *Brassica napus* (Mehmood et al., 2021) and *Brassica juncea* (Sinha et al., 2015). Microarray analysis of peanut leaves subjected to low temperature got a number of cold-regulated genes and provided some useful insights into cold signal transduction pathways in peanut (Chen et al., 2014). With the rapid development of high-throughput sequencing and mass spectrometer technologies, a massive of multi-omics data (e.g., transcriptomics and metabolomics) have been generated, and it is possible to explore the mechanism of plant cold tolerance by analyzing and mining the big data (Zhao et al., 2019; Cui et al., 2020; Du et al., 2020; Yang et al., 2020; Jiang et al., 2021). Comparative transcriptomic analysis of two peanut varieties with contrasting cold resistance (NH5 and FH18) identified several cold-responsive transcription factors, including bHLH, MYB, and NAC (Jiang et al., 2020). Furthermore, lipidomic analysis of the two varieties revealed that membrane lipid and fatty acid metabolisms contributed substantially to cold resistance in peanut (Zhang H. et al., 2020).

Although there are a few reports about cold tolerance in peanut, the molecular mechanism underlining the signal pathway and related gene networks need to be further investigated due to the complexity of the cold-resistant trait in this important crop (Wang et al., 2017, 2020; Zhang et al., 2019). In the current study, an integrated analysis of transcriptome and metabolome from two peanut cultivars (SLH and ZH12) allowed us to obtain a set of cold-responsive metabolites/genes that possibly protect peanut from chilling damage. These results contributed to a better understanding of the molecular mechanism of cold response in peanut and will be useful for the development of cultivars with enhanced cold-stress tolerance.

## Materials and Methods

### Plant Materials and Treatments

The cold-resistant peanut cultivar “Silihong” (SLH) and cold-sensitive “Zhonghua 12” (ZH12) were used in this study. Peanut seeds were soaked in distilled water for 4 h, and placed in petri dishes with moistened filter papers, and then germinated in the dark at 28°C for 2 days. The germinated seeds were sown in soil pots and transferred into a growth chamber under a 16-h/8-h (light-dark) cycle at 28°C, with a photosynthetic photon flux density of 700 μmol m^–2^ s^–1^. Two-week-old seedlings were used for cold treatment with a 16-h/8-h cycle (light/dark) at 10°C. The third leaves from seedlings were collected at 0, 3, 24, and 48 h, quickly frozen in liquid nitrogen, and stored at −80°C until use. All samples were performed in three independent biological replicates.

### Physiological Index Measurements

The two varieties of peanut seeds were immersed in distilled water for 4 h, transferred to petri dishes at 2°C for 72 h, and then returned to normal temperature (28°C) for additional 3 days. The controls were continuously germinated at 28°C for 3 days. The germination rates were measured every day.

Malondialdehyde (MDA) and H_2_O_2_ content were determined by Lipid Peroxidation MDA Assay Kit and Hydrogen Peroxide Assay Kit (Beyotime Biotechnology Co., Ltd., Shanghai, China), respectively. The superoxide anion level was analyzed by Micro Superoxide Anion Assay Kit (Beijing Solarbio Science and Technology Co., Ltd., Beijing, China). All the experiments were performed according to the manufacturers’ protocols, and data were derived from three biological replicates. Student’s *T-*test was performed to calculate the *p*-values using SPSS 20.0 version.

### Metabolite Extraction and Profiling

The frozen leaves were grounded into fine powder in liquid nitrogen. About 100 mg of samples was placed into 2-ml Eppendorf tubes and resuspended with 500-μl extraction liquid (80% methanol and 0.1% formic acid). After vortex for 30 s, the mixtures were incubated on ice for 5 min and then centrifuged at 15,000 rpm, 4°C for 10 min. Some of the supernatant was transferred to a fresh EP tube and diluted to final concentration containing 53% methanol by adding with LC-MS grade water. The samples were centrifuged at 15,000 *g*, 4°C for 20 min. Finally, the supernatant was injected into the LC-MS/MS system for analysis.

For the positive polarity mode, LC-MS/MS analyses were performed using an ExionLC^TM^ AD system (SCIEX) on a BEH C8 Column (100 mm × 2.1 mm, 1.9 μm), coupled with a QTRAP^®^ 6500 + mass spectrometer (SCIEX). The mobile phase consisted of 0.1% formic acid water (A) and acetonitrile (B) with elution gradient at a flow rate of 0.35 ml/min as follows: 5% B, 1 min; 5–100% B, 24 min; 100% B, 28 min; 100–5% B, 28.1 min, 5% B, 30 min. For the negative polarity mode, samples were injected onto an HSS T3 Column (100 mm × 2.1 mm). The flow rate was set as 0.35 ml/min with solvent gradient as follows: 2% B, 1 min; 2–100% B, 18 min; 100% B, 22 min; 100–5% B, 22.1 min; 5% B, 25 min. The main parameters of QTRAP^®^ 6500 + mass spectrometer were set as follows: Curtain Gas of 35 psi, Collision Gas of Medium, Temperature of 500°C, Ion Spray Voltage of 5,500 V or −4,500 V in a positive or negative mode, respectively.

Metabolite identification was based on the fragmentation patterns of Q1 and Q3, retention time, decluttering potential, and collision energy. Q3 was used to quantify the metabolite according to the in-house database (Novogene Bioinformatics Technology Co., Ltd.).

### Transcriptome Sequencing

Total RNA was isolated from the peanut leaves. The concentration and integrity of RNA were checked by the Bioanalyzer 2100 system (Agilent Technologies, CA, United States). About 1 μg of RNA per sample was used as input material for the transcriptome library preparation. The libraries were sequenced on an Illumina NovaSeq platform (Tianjin, China), and 150-bp paired-end reads were generated. The high-quality reads were obtained by removing reads containing adapter, reads containing poly-N, and low-quality reads from raw data. The high-quality paired-end reads were then mapped to the peanut reference genome (Tifrunner.gnm1.ann1.CCJH^1^) using HISAT2 software (version 2.0.5) with default parameters (Kim et al., 2019). The feature counts program (with a parameter of v1.5.0-p3) (Liao et al., 2014) was used to count the reads numbers mapped to each gene. If a read was mapped to two or more positions in the genome, then it will be filtered out. Only uniquely mapped reads were used for quantification. The gene expression level was calculated by the FPKM values (Fragments per Kilobase of transcript per Million mapped reads) using the Cufflinks program (Trapnell et al., 2010). The Pearson correlation coefficients between different samples were calculated by the cor function in R package and presented as heatmap using pheatmap software^2^. To identify cold-responsive genes in peanut, the differentially expressed genes (DEGs) were screened using the DESeq2 R package (1.20.0) with the criteria of | log2FC (fold change) | > 1 and an adjusted *P*-value < 0.01 (Love et al., 2014). Gene ontology (GO) and KEGG enrichment analysis of DEGs were performed by the cluster Profiler R package (Yu et al., 2012).

### Analysis of Gene Expression by Real-Time Quantitative RT-PCR

Total RNA was extracted from the peanut leaves of three replicates using an EASYspin plant RNA extraction kit (Aidlab Biotechlogies, Co., Ltd., China) according to the manufacturer’s instructions. First-strand cDNA was synthesized from DNase I-treated total RNA (about 1 μg) using an MMLV reverse transcriptase kit (Thermofisher Scientific, United States). Quantitative RT-PCR (qRT-PCR) assays were performed on the Bio-Rad CFX96 RT-PCR Detection system (Bio-Rad, Hercules, CA, United States) using Hieff qPCR SYBR Green Master Mix (YEASEN, Shanghai, China). The relative transcript levels among different samples were quantified by the 2^–ΔΔCt^ method (Livak and Schmittgen, 2001), using *A. hypogaea* Actin gene (accession number: Aradu.W2Y55) as a reference gene for normalization. The expression level of 0 h in SLH was used as a control, whose value was set to 1. The relative expression levels of other samples (including a 0-h time point in ZH12) were determined relative to the control. In this way, we can compare all samples across the two varieties within the same gene. The information of genes for qRT-PCR analysis is listed in Supplementary Table 1.

### Weighted Gene Co-expression Network Analysis

Weighted gene co-expression network analysis (WGCNA) can be used for identifying genes with similar expression patterns that may participate in specific biological functions (Langfelder and Horvath, 2008). A total of 3,620 cold tolerance core genes were used as an input in the R package WGCNA (version 1.47) to construct weighted co-expression modules with following parameters: weighted network = unsigned, power = 10, minimum module size = 30, minimum height for merging modules = 0.25. Then, the calculated module eigen genes and Pearson’s correlation coefficient values were used to determine the association of modules with the metabolite contents (soluble sugars, polyamines, amino acids, and phenols) for the 24 samples. The number of edges in a node represented the hubness of the gene. Hub genes were ranked based on the module eigengene values and edge numbers, and they often exhibit the most connections with other genes within a module. The gene networks and top 20 hub genes within a module were visualized by Cytoscape software (Shannon et al., 2003).

## Results

### Physiological Differences Between SLH and ZH12 in Response to Cold Stress

**FIGURE 1:**
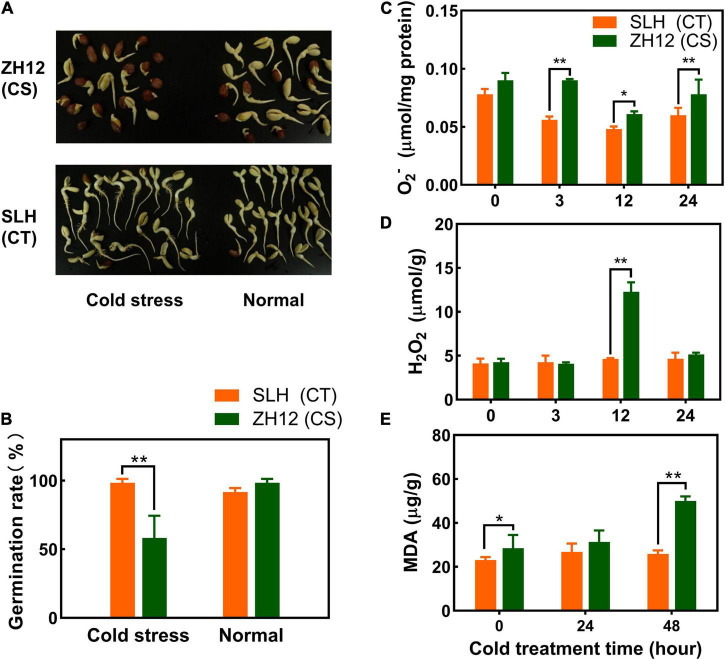
Differential physiological responses of SLH and ZH12 peanut cultivars to cold stress. **(A,B)** Effects of low temperature on germination rates of the two peanut cultivars. For cold treatment, seeds were immersed in distilled water for 4 h, and then transferred to petri dishes at 2°C for 72 h in the dark, and, finally, returned to normal temperature (28°C) for additional 3 days for germination. The controls were continuously germinated at 28°C for 3 days. SLH (CT), the cold-tolerance cultivar; ZH12 (CS), the cold-susceptible cultivar. The level of O_2_^–^ **(C)**, H_2_O_2_ **(D)**, and malondialdehyde [MDA, **(E)**] of peanut seedlings in response to chilling stress. Error bars represent the SD of the means of three biological replications. *An asterisk indicates significant difference between the two cultivars as determined by the Student’s *T-*test (*p < 0.05; **p < 0.01).

Two peanut cultivars with differential cold tolerance (SLH and ZH12) were selected for investigating their physiological traits in response to chilling stress (Zhang et al., 2021). SLH has been characterized as a cold-tolerant germplasm, and it is popularly planted in northeast China (a relative high latitude region, 48°N–55°N) where cold weather often happens in the early sowing and harvest seasons. ZH12 (cold susceptible) is a representative variety released by Oil Crops Research Institute, Chinese Academy of Agricultural Sciences, and it is commonly planted in Hubei province, central China (a low latitude region, 29°N–33°N), which has a warm climate in the sowing and harvest season. It was found that the two cultivars showed similar germination rates under normal condition but were severely affected for ZH12 in comparison to SLH when they were exposed to low temperature (Figures 1A,B). Additionally, the levels of hydrogen peroxide (H_2_O_2_) and superoxide anion (O_2_^–^) were significantly higher in the seedlings of ZH12 than those of SLH within 24 h of cold treatment (10°C) (Figures 1C,D). It is known that ROS overproduction brings about lipid peroxidation, and the malondialdehyde (MDA) level is an important indicator for membrane damage (Jambunathan, 2010). In the current study, the leaf MDA contents in cold-treated ZH12 increased about 2-fold compared with control (0 h) and displayed higher levels than those of SLH after 48-h cold treatment (Figure 1E). The pronounced enhancement of MDA content in susceptible peanut cultivar ZH12 was presumably caused by excessive accumulation of O_2_^–^ and H_2_O_2_ under chilling stress. Thus, the difference of physiological responses to low temperature confirmed that SLH was relatively stable, while ZH12 was more susceptible to cold damage.

### Metabolome Profiling of Peanut in Response to Cold Stress

**FIGURE 2:**
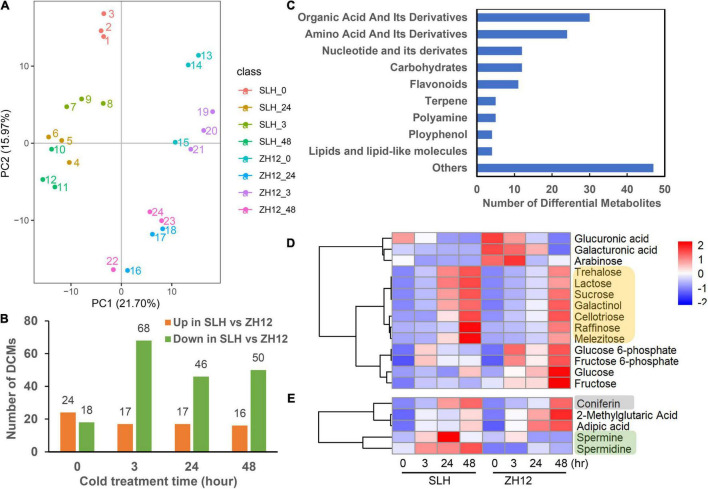
Metabolite profiling of two peanut varieties exposed to cold stress. **(A)** Principle component analysis (PCA) of metabolome data derived from SLH and ZH12 during four time points under cold stress (0, 3, 24, 48 h, with three biological replicates per point). **(B)** The differentially changed metabolites (DCMs) between the SLH and ZH12 under cold stress. The DCMs were screened by the criteria of | log_2_ (fold change) | > 1 and *P*-value < 0.05. **(C)** Classification of 145 DCMs between the two cultivars. **(D,E)** The accumulation pattern of carbohydrates (indicated by yellow), polyamines (marked by green), and phenols (gray) under cold stress in SLH and ZH12. The relative contents of metabolites (represented by the LC-MS/MS peak area) in different samples were used for z-scale normalization in the heatmap. The red color indicated relative high content of substances, while blue was low.

To investigate metabolome profiling of the two peanut genotypes exposed to low temperature, leaves were collected at the four different treatment time points (with three biological replicates per point), and further subjected to UPLC-MS for analyzing their metabolic changes. As a result, a total of 563 metabolites were successfully detected, and these could be mainly classified into different categories, including amino acids, carbohydrates, nucleosides, lipids, alkaloids, flavonoids, terpenoids, etc., (Supplementary Table 2). Principle component analysis (PCA) of metabolites profiling showed that the two cultivars were separated by PC1 (21.70%), and samples collected at different time points were separated by PC2 (15.97%) (Figure 2A). Moreover, the control and cold-treated samples were separated, indicating that low temperature had profound impacts on the compound accumulation patterns in peanut.

To identify the metabolites that contribute to cold tolerance in peanut, we performed comparative analysis of the metabolomic changes between SLH and ZH12. Using thresholds for | log_2_ (fold change) | > 1 and *P*-value < 0.05, a total of 42, 85, 63, and 56 differentially changed metabolites (DCMs) were found between the two cultivars in the four time points of cold treatment, respectively (Figure 2B and Supplementary Table 3). Altogether, we identified 145 DCMs between the two cultivars, which were mainly involved in organic acid, amino acids, nucleotides and carbohydrates (Figure 2C and Supplementary Table 4). Among the changed carbohydrates, the contents of seven sugars (e.g., raffinose, trehalose, lactose, galactinol, cellotriose, melezitose, and sucrose) were found to be significantly increased under cold stress. Moreover, it appeared that SLH exhibited at an earlier induction time point (at 24 h) and displayed more enhanced levels of the sugars compared to ZH12 in response to low temperature (Figure 2D and Supplementary Table 5). Similarly, other metabolites, such as polyamines (spermine, spermidine) and phenols (coniferin), also exhibited higher accumulation patterns in SLH than ZH12 (Figure 2E). As some soluble sugars, polyamines and phenols were reported to be involved in plant cold response (Fuertauer et al., 2019; Alcazar et al., 2020), the differential accumulation patterns of these substances probably provided stronger cold tolerance capability in SLH.

### Transcriptomic Analysis of Peanut Exposed to Cold Stress

#### General Description of Transcriptome Data

**FIGURE 3:**
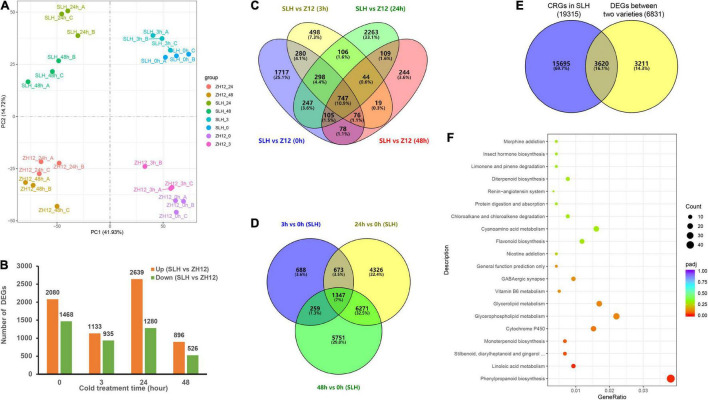
Transcriptome analysis of two peanut cultivars in response to cold stress. **(A)** Principle component analysis (PCA) of transcriptome data derived from SLH and ZH12 during four time points under cold stress (0, 3, 24, 48 h, with three biological replicates per point). **(B)** The number of differentially expressed genes (DEGs) between SLH and ZH12 exposed to cold treatment. DEGs were filed out using the DESeq2 R package with the parameter of | log_2_ (fold change)| > 1 and an adjusted *P*-value < 0.01. **(C)** The Venn diagram of DEGs between the two cultivars at four different cold treatment time points. **(D)** The Venn diagram of Cold Responsive Genes (CRGs) in SLH in the three cold exposure times (3, 24, and 48 h) compared to control (0 h). **(E)** The Venn diagram showing 3,620 putative Cold Tolerance Genes (CTG) in peanut. The yellow circle refers to the total 6,831 DEGs during the cold treatments between the two cultivars as shown in panel **(C)**, and the blue circle represents the total 19,315 cold-responsive genes (CRGs) in SLH as indicated in panel **(D)**. **(F)** KEGG enrichment analysis of the 3,620 Cold Tolerance Genes (CTG) in peanut.

Total mRNA extracted from peanut leaves was subjected to construct sequencing libraries using the Illumina paired-end platform. With three biological replicates per time point, the 24 samples yielded more than 185.44 Gb high-quality data with an average Q30 score of 93.09% (Supplementary Table 6). A total of 48,374 and 46,844 unigenes were obtained from SLH and ZH12 with FPKM values > 0.1 in at least one time point, respectively (Supplementary Figures 2A,B). There were 38,741 and 37,705 transcripts expressed at all-time points in SLH and ZH12, respectively. Over 90% (45,302) of the detected transcripts were shared by the two genotypes (Supplementary Figure 2C). Pearson correlation analysis of RNA-seq data displayed high reproducibility between biological replicates (Supplementary Figure 3). PCA analysis indicated that the samples from the different time points could be separated by the PC1 (41.93%), and the genotypes were separated by the PC2 (14.72%) (Figure 3A). Additionally, the number of cold-responsive genes in 24 or 48 h is greater than that in 3 h (Supplementary Figure 4). This suggested that prolonged duration of cold stress resulted in more profound changes of a transcriptome profile in peanut.

#### Identification of Putative Cold Tolerance Genes in Peanut

**FIGURE 4:**
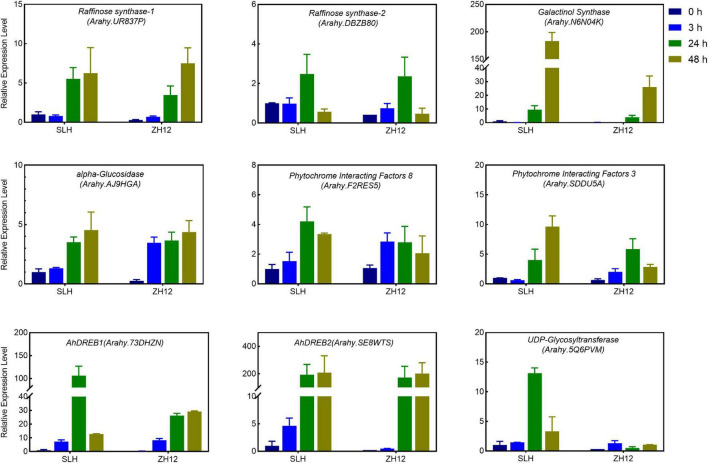
Quantitative RT-PCR (qRT-PCR) analysis of the expression levels of selected Core Cold Tolerance Genes in the peanut seedlings subjected to cold stress. The relative transcript levels among different samples were quantified by the 2^− ΔΔCt^ method, with *A. hypogaea* Actin gene as the reference gene for normalization. Error bars indicate the S.D. of the means of three biological replicates.

To get a deep insight into the mechanism of cold tolerance in peanut, we performed comparative analysis of the transcriptional difference between SLH and ZH12 at the four time points of cold treatment (Figures 3B,C). In total, 6,831 differentially expressed genes (DEGs) were found between the two contrasting cultivars (Supplementary Table 7). Since genes involved in cold tolerance were probably induced and/or depressed by low temperature, we identified a total of 19,315 cold-responsive genes (CRGs) in SLH by comparing the transcriptome profile in 3, 24, and 48 h with that of control (0 h) (Figure 3D and Supplementary Table 8). As a result, there were 3,620 common genes shared by DEGs between the two cultivars and cold-responsive genes in SLH (Figure 3E). These common genes were believed to be a putative “Cold Tolerance Gene set” (CTGs) in peanut (Supplementary Table 9). Several transcription factors that were known to be involved in the regulating cold-signaling pathway were included in CTGs, such as two DREBs (Arahy.73DHZN and Arahy.SE8WTS) and two phytochrome interacting factors (Arahy.SDDU5A and Arahy.F2RES5) (Figure 4). Also, some structure genes in the carbohydrate metabolism were found in CTGs, including two raffinose synthases (Arahy.UR837P and Arahy.DBZB80) and a galactinol synthase (Arahy.N6N04K). The gene expression changes of nine CTGs were analyzed by qRT-PCR. Their transcript levels were higher and/or induce a data earlier time point by cold stress in SLH (cold tolerant) than ZH12 (cold susceptible), which had a good correlation with RNA-seq results (Figure 4 and Supplementary Figure 5).

#### Functional Enrichment Analysis of Cold Tolerance Genes in Peanut

Based on the GO enrichment analysis, the 3,620 CTGs were distributed into different known GO terms (Supplementary Table 10 and Supplementary Figure 6). Among the “biological process” category, the most significantly enriched terms are “response to biotic stimulus” and “defense response.” In addition, the “metal ion transport” term included 14 genes encoding for heavy metal-associated isoprenylated plant proteins (HIPPs), which were reported to play important roles in response to biotic/abiotic stresses (Zhang et al., 2015). The top enriched GO terms in “molecular functions” were related to “transferring acyl groups” and “glucosyltransferase activity.” Of these terms, 12 genes were annotated as putative cellulose synthases involved in the biosynthesis of the hemicellulose backbone, which might be helpful to confer abiotic stress tolerance to plants (Endler et al., 2015; Huang et al., 2021; Yuan et al., 2021).

KEGG pathway enrichment analysis of CTGs showed that the top significant enriched pathways were “phenylpropanoid biosynthesis” and “linoleic acid metabolism,” and “stilbenoid, diarylheptanoid, and gingerol biosynthesis” (Figure 3F and Supplementary Table 11). As one of the major branches of the phenylpropanoid pathway, lignin biosynthetic genes were upregulated by cold stress. In addition, the “linoleic acid metabolism” pathway included 11 lipoxygenase genes that were known to be related cold acclimation in plants (Upadhyay et al., 2019). These results indicate that both lignin and lipids take a great part in response to cold stress in peanut.

#### Identification of Key Genes and Modules in Response to Cold Stress by Weighted Gene Co-expression Network Analysis

**FIGURE 5:**
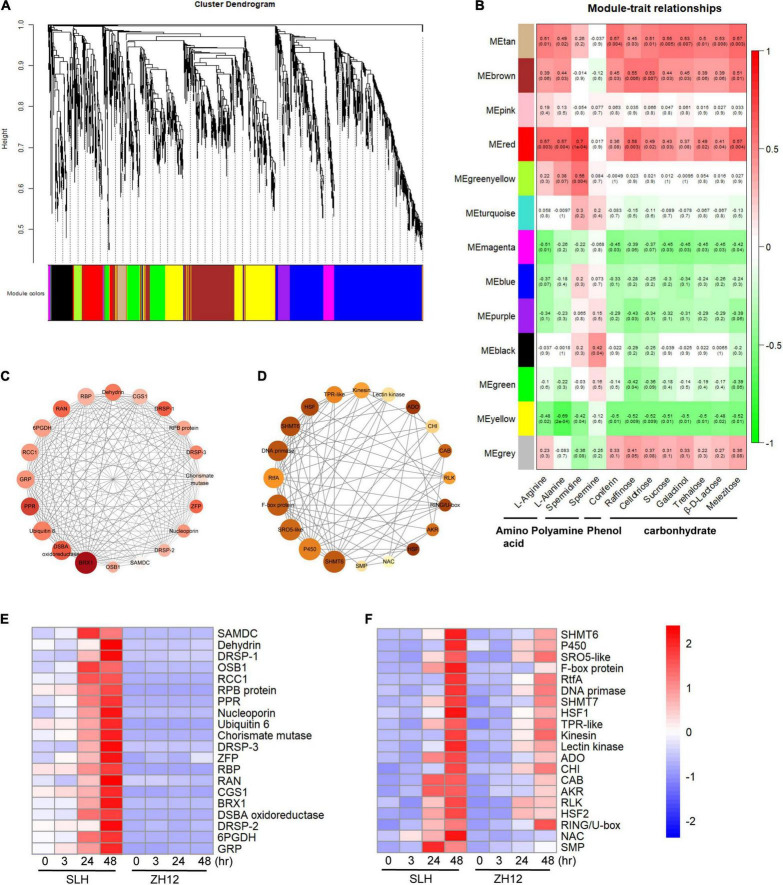
Identification of hub genes and key modules responsive to cold stress in peanut by WGCNA. **(A)** The hierarchical cluster tree shows 13 modules of co-expressed genes. Different modules are marked with different colors. **(B)** Correlations of metabolite contents with WGCNA modules. Each row corresponds to a specific module eigengene and a column to a trait. The values in each cell represent the correlation coefficients (r), and the *p*-values (in parentheses) of the module-trait association. The deeper the red or green in a cell, the higher the positive or negative correlation between the module eigengene and a trait feature. Visualization of the key co-expression network red module **(C)** and the tan **(D)** module by Cytoscape. The top 20 hub genes with the highest degree of connection to other genes are shown in the nodes. The color of node corresponds to degree value, and size means module membership value calculated by WGCNA. The darker and larger of the nodes indicates the greater hubness of the gene. Hierarchical cluster analysis of the top 20 hub genes in the red module **(E)** and the tan **(F)** module. The values in the heatmap **(E,F)** represent the z-score of the FPKM (transcript levels) in different samples. Red color indicates a high expression level, while blue is low.

Weighted gene co-expression network analysis is a popular systems biology method used to not only construct gene networks but also detect gene modules and identify the central players (i.e., hub genes) within modules. To obtain a comprehensive understanding of the molecular mechanism of cold tolerance in peanut, the 3,620 CTGs were put into the WGCNA software R package to build a gene co-expression network (Langfelder and Horvath, 2008). Based on pairwise correlations analysis of gene expression, 13 merged co-expression modules marked with different colors are shown in Figure 5A and could be further clustered into two main branches (Figure 5A and Supplementary Table 12). Analysis of the module-trait relationships for the 24 samples revealed that amino acids (Arginine and Alanine), spermidine, and two sugar compounds (raffinose and melezitose) were tightly associated with a red module (*R* > 0.5 and *p* < 0.01), while coniferin and the other three soluble sugars (trehalose, glucose, and sucrose) were more related to the tan module (Figure 5B). Based on the values of WGCNA edge weight and node scores, the top 20 hub genes were identified in the red module (Supplementary Table 13). These genes encoded some important proteins involved in abiotic stress processes, including dehydrin (arahy.4JU6QM), 6-phosphogluconate dehydrogenase (6PGDH, arahy.RH103U), pentatricopeptide repeat family protein (PPR, arahy.97P92R), nucleoporin (arahy.9L03M9), and *S*-adenosylmethionine decarboxylase (SAMDC, arahy.0034KJ) and GTP-binding nuclear protein (Ran, arahy.G34P0I) (Figure 5C). For the tan module, the top 20 genes encoded transcription factors (such as heat shock transcription factor and NAC-domain protein) and several stress-related proteins/enzymes, including cytochrome P450 (P450, arahy.MG688V), serine hydroxy-methyltransferase 6 (SHMT6, arahy. HK9TK1), F-box protein (arahy.C9YQVF), and SRO5 (arahy.7WJT5G) (Figure 5D and Supplementary Table 13). Notably, the transcript levels of hub genes in both of the modules were remarkably upregulated in SLH but were hardly expressed or only slightly enhanced in ZH12 at a late treatment point (48 h) (Figures 5E,F).

### Integrated Metabolome and Transcriptome Analysis to Reveal Crucial Pathways Responsive to Cold Stress

**FIGURE 6:**
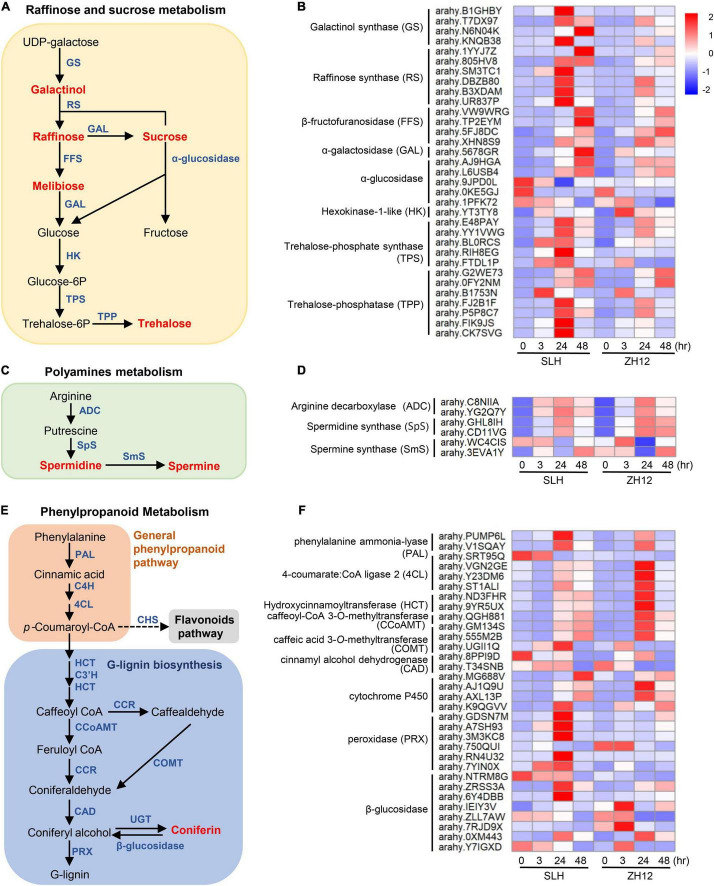
Integrated analysis of metabolomic and transcriptomic profiles of peanut under cold stress. The schematic diagrams of raffinose and sucrose **(A)**, polyamines **(C)**, and phenylpropanoid **(E)** metabolism pathways. In the diagrams, the metabolites are marked by red, and structural enzymes are indicated by blue. The transcriptional patterns of several structural genes involved in the raffinose and sucrose **(B)**, polyamines **(D)**, and phenylpropanoid **(F)** metabolism pathway under cold stress. The heatmap values represent the Z-score of the FPKM (transcript levels) in different samples. Red color indicates a high expression level, while blue is low.

An integrated analysis of transcriptome and metabolome data revealed some common enriched pathways, including “carbohydrates metabolism” and “polyamines metabolism.” For carbohydrates metabolism, most of raffinose family oligosaccharides (RFOs) and sucrose-related metabolites, such as galactinol, raffinose, melibiose, and trehalose, were markedly increased in the two genotypes under cold stress. It should be noted that the fold changes of sugar compounds were consistently higher in cold-tolerant (SLH) than in cold-susceptible genotypes (ZH12), suggesting their vital roles in protection of peanut against low temperature stress (Figures 2C, 6A). Accordingly, the transcriptional profile of most genes involved in sugar biosynthetic pathways correlated well with the accumulation pattern in the two cultivars exposed to cold stress (Figures 2D, 6B). For instance, four genes-encoding galactinol synthases (EC 2.4.1.123, which catalyze the key steps for galactinol synthesis (Sengupta et al., 2015; Shimosaka and Ozawa, 2015), were greatly upregulated in SLH, but their transcripts were not changed or only slightly upregulated under cold stress in ZH12. Raffinose synthase, also called as galactinol-sucrose galactosyltransferase (EC 2.4.1.82), was a rate-limiting enzyme in biosynthesis of raffinose (ElSayed et al., 2014). Interestingly, expression levels of all the six raffinose synthase were strong cold induced in SLH, while moderately enhanced in ZH12. Besides, most of the genes involved in the biosynthesis pathways of sucrose, melibiose, and trehalose were upregulated in both genotypes in response to low temperature, but their expression levels increased more significantly in SLH than in ZH12. These results suggested that the cold tolerance of SLH might be associated with its stronger ability to regulate carbohydrates metabolism, thus leading to a higher accumulation of sucrose, raffinose, and trehalose in SLH than in cold-susceptible genotype (ZH12).

Polyamines, including putrescine, spermidine, and spermine, are aliphatic nitrogen substances with low molecular weight and polycation characteristics, which have important roles in improving abiotic stress tolerance, including low-temperature tolerance (Alcazar et al., 2011). Several key enzyme genes involved in the “spermidine biosynthetic process,” including spermidine synthase (E.C. 2.5.1.16) and spermine synthase (E.C. 2.5.1.22), were found to be upregulated in response to cold stress (Figures 6C,D). This was in accordance with the accumulation pattern of polyamines in both peanut cultivars. Moreover, as compared with susceptible peanut (ZH12), the cold-tolerant one (SLH) had higher cellular levels of spermidine and spermine, suggesting it has a larger capacity to enhance polyamine biosynthesis under low temperature.

It has been reported that lignin serves as one of the major contributors to abiotic stress resistance (Cesarino, 2019). Coniferin, the glucoside of coniferyl alcohol, was considered as the most possible storage form of monolignol for G-lignin biosynthesis, which helps plants to fight against several abiotic stresses (Tsuji et al., 2005; Sharma et al., 2019). In this study, cold stress greatly increased coniferin contents in the peanut seedlings (Figures 2E, 6E). Accordingly, several genes in the general phenylpropanoid pathway and downstream G- lignin biosynthesis were significantly upregulated, including two phenylalanine ammonia lyases (PALs, the entry enzyme for phenylpropanoids), four 4-coumarate-CoA ligases (4CLs), two caffeic acid 3-*O-*methyltransferases (COMTs), and one caffeoyl-CoA 3-*O*-methyltransferase (CCoAOMT) (Figure 6F and Supplementary Table 12). However, the transcript levels of these genes showed a differential expression pattern between the two cultivars. For instance, two *PALs* and one *COMT* gene had significant higher transcript levels in SLH than in ZH12, while the expression levels of four *4CLs*, two hydroxycinnamoyltransferases (*HCTs*), and a*CCoAOMT* gene were higher in ZH12 compared to SLH after 24-h cold treatment (Figure 6F).

## Discussion

Cold weather accounts for serious reductions in crop yield every year, since many agriculturally important crops, including rice (*Oryza sativa*) and peanut, are chilling sensitive and can only be cultivated in tropical or subtropical regions (Wu et al., 2020). Despite the molecular mechanisms of cold-induced reprogramming of gene expression and the metabolite accumulation pattern were extensively studied in model plants (Hoermiller et al., 2016; Jin et al., 2017; Pagter et al., 2017; Dasgupta et al., 2020), only a handful of reports were related to peanut (Jiang et al., 2020; Wu et al., 2020; Zhang H. et al., 2020). In this study, we attempted to decipher the regulatory mechanisms of peanut in response to cold stress through combining transcriptome with metabolome analysis.

### Analysis of Signal Transduction and Protein Kinases in Response to Cold Stress

Ca^2+^ is an important signaling messenger in response to cold stress (Yuan et al., 2018). The calmodulins (CAMs) and calcium-dependent protein kinases (CDPKs) are vital calcium sensors and could transduce cold signals to activate the expression of downstream cold-response gene (*CORs*), including *DREBs/CBFs* and other components (Yang et al., 2010; Zeng et al., 2015). Overexpression of a salt-induced peanut *CDPK* gene in tobacco could alleviate PSII photoinhibition under abiotic stress, suggesting it played an important role in stress tolerance in peanut (Li et al., 2015). In the current study, the transcription of three calmodulin-binding proteins (CBPs, Arahy.1Y8NUF, Arahy.R2NPVK, and Arahy.GM51F1) and four CDPKs (Arahy.706LQI, Arahy.D2QNFF, Arahy.D2U3E6, and Arahy.P2RW1V) were significantly induced by low temperature (Supplementary Figures 7A,B and Supplementary Table 14) and have a relative higher transcript abundance in SLH (cold tolerant) than ZH12. Also, a total of 17 *CBF* transcripts were identified in our RNA-seq database (Supplementary Table 15). Among them, seven *CBFs* were not expressed at any time points in both of the varieties, while most of the other transcripts were increased in response to cold treatment (Supplementary Figure 7). It should be noted that some of the upregulated *CBFs* (arahy.73DHZN, arahy.WP3ECB, and arahy.5KI8FH) were induced at an earlier time point and/or have relative higher expression levels in cold tolerance variety (SLH) than that in cold susceptible (ZH12) (Figure 4 and Supplementary Figure 7), implying their significance in cold-signal perception and transduction processes in peanut.

In the present study, the transcript expression of two Heat Shock transcription Factors (HSFs) (Arahy.8U41WB and Arahy.W19Z82) was induced by cold stress in both cultivars (Figure 5F), and their transcript levels exhibited much higher in the cold-tolerant (SLH) than susceptible cultivar (ZH12) at 24- or 48-h treatment. Plant HSFs are encoded by large gene families with variable structure, expression, and function. They control responses not only to high temperatures but also to a number of abiotic stresses, such as cold, drought, and salt stress (Andrási et al., 2021). Some reports have shown that transcript abundances of *HSF* (*HSFA4A*, *HSFA6*B, *HSFA8*, and *HSFC1*) were enhanced by cold stress in *Arabidopsis*, while the induction effect was reduced in the *ice1* mutant, implicating HSFs were involved in the cold-acclimation pathway (Swindell et al., 2007; Olate et al., 2018). In addition, overexpression of HSFs can stimulate the synthesis of protective metabolites, such as galactinol and raffinose, to improve abiotic stress tolerance in plants (Nishizawa et al., 2006; Lang et al., 2017). Therefore, it was proposed that the relative higher transcript levels of *HSFs* in SLH could be more beneficial for it to cope with cold stress.

### Carbohydrate Metabolism Contributes Greatly to Cold Tolerance in Peanut

Carbohydrates are the primary products of photosynthesis. They act as nutrients as well important regulators that are required for plant growth, energy metabolism, and stress responses (Fuertauer et al., 2019). It was known that soluble sugars can not only function as osmoprotectants (Zuther et al., 2004; Lunn et al., 2014; Ma et al., 2021) but also to be ROS scavengers to provide cold tolerance in plants (Smirnoff and Cumbes, 1989; Van den Ende, 2013; Matros et al., 2015). In our study, the concentrations of soluble sugars (e.g., sucrose, trehalose, and raffinose) greatly raised in the two peanut cultivars exposed to cold stress. Notably, their contents were higher and/or induced at an earlier time point in cold-resistant peanut (SLH) than those in susceptible cultivar (ZH12) (Figure 2D). It has been reported that chilling stress could lead to a significant increase in H_2_O_2_ concentration in peanut (Wu et al., 2020). Similarly, we found that low temperature caused a sudden increase in the H_2_O_2_ in ZH12 rather than in SLH after 12-h treatment. Thus, it was proposed that the higher accumulation of soluble sugars probably led to maintain a low level of ROS in the cold-tolerant cultivar (SLH), which facilitated to its relative stronger cold-tolerance ability than the susceptible one (ZH12). On the other hand, ROS (including O_2_^–^ and H_2_O_2_) can be detoxified by enzymatic and non-enzymatic antioxidant systems to maintain them at non-toxic levels in a delicate balancing state. In our transcriptome data, the transcript levels of several antioxidant enzymes (including superoxide dismutase and peroxidase) were enhanced in ZH12 (and also in SLH) during the early cold treatment period (3 and 24 h) (Supplementary Figures 7D,E). Thereby, we hypothesize that the upregulation of these antioxidant enzymes might efficiently scavenge the excess H_2_O_2_, rendering it recovered to the normal level (0 h) in 24 h.

In addition, the accumulation patterns of raffinose and sucrose between the two cultivars correlated well with expression profiles of their structural enzyme genes in the pathway (Figures 2D,E, 6A,B). Several studies have highlighted the role of these genes in cold stress (Lunn et al., 2014; Sengupta et al., 2015). For instance, overexpression of cold-inducible wheat galactinol synthase increased levels of galactinol and raffinose, and conferred higher tolerance to chilling stress in transgenic rice (Shimosaka and Ozawa, 2015). Simultaneously, exogenous application of sucrose improved chilling tolerance in cucumber seedlings by increasing antioxidant enzyme activity, and enhanced proline and soluble sugar contents. These results indicated that genes and metabolites involved in carbohydrate metabolism contributed much to cold tolerance in peanut.

### Polyamine Metabolism Plays Central Roles in Cold Resistance in Peanut

Several studies have shown that the levels of polyamine were substantially elevated in plants exposed to stressful conditions, including drought, salinity, chilling, and heat (Chen et al., 2019). The improvement of cold-stress tolerance could be achieved by exogenous application of polyamines or genetic manipulation of endogenous polyamine levels (Kou et al., 2018; Alcazar et al., 2020). In the present study, spermine and spermidine were specifically accumulated in SLH (cold resistant) under chilling stress. Meanwhile, transcriptomic and WGCNA analysis showed that several transcripts and/or hub genes in polyamines biosynthesis have a relatively higher level in SLH than ZH12. For instance, *S*-adenosylmethionine decarboxylase (SAMDC, arahy.0034KJ), a key enzyme in the polyamine biosynthetic process, was identified as one of the hub genes in the red module (Figures 5C,E). It has been reported that overexpression of a bermudagrass SAMDC (*CdSAMDC1*) gene in tobacco enhanced plant cold tolerance through involvement of H_2_O_2_ and NO signaling (Luo et al., 2017). Like the transcriptional patterns of other hub genes in this module, the expression level of SAMDC was increased upon prolonged cold stress in SLH, but was not induced in ZH12 samples. These results suggested polyamine metabolism played crucial roles in cold resistance in peanut.

### G-Lignin Biosynthesis Is Involved in Cold-Stress Response

Phenylpropanoid biosynthesis is one of the most important metabolisms in plants, generating an enormous array of secondary metabolites, such as lignin and flavonoid (Dong and Lin, 2021). Recent studies have revealed that cold stress induced the expression of structural genes in the phenylpropanoid pathway, including chalcone synthase (CHS) and 4-coumarate-CoA ligase (4CL), and, as a consequence, flavonoids and lignin accumulated to facilitate the adaptation to low-temperature environments in *A. thaliana* (Catalá et al., 2011), loquat fruit (*Eriobotrya japonica*) (Zhang J. et al., 2020), and apple (*M. domestica*) (An et al., 2020). In the current study, we found that some cold-responsive genes involved in the common phenylpropanoid metabolism and downstream G-lignin biosynthesis showed higher expression levels in cold-tolerant cultivar (SLH) than those in ZH12 (Figure 6F). For instance, PAL is the rate-limiting enzyme that catalyzes the deamination of L-phenylalanine to *trans*-cinnamate and forms the entry point into the synthesis of all phenylpropanoids. The higher transcript level of two PALs in SLH compared with those in ZH12 could drive more carbon flux into the phenylpropanoid pathway, probably leading to more coniferin accumulation and better cold tolerance in SLH.

## Conclusion

In the present study, combining metabolomic and transcriptomic analysis of peanut seedlings exposed to chilling stress identified a set of important cold-responsive genes and metabolites, some of which showed differential expressions and/or accumulation patterns between the two cultivars. The signaling molecule Ca^2+^ and its related components first sense the cold stress, and then activate downstream pathways. During the adaptation of peanut to low temperature, soluble sugars, polyamines, and G-lignin might be the significant contributors. These results facilitate a better understanding of the molecular mechanism of cold response in peanut and could be helpful to genetic improvement of cold tolerance of crops. So far, we have heterogeneously overexpressed several peanut cold-tolerance-related genes in *Arabidopsis*, and obtained some transgenic lines. We are now analyzing their phenotypes under normal and stressful conditions. However, this part has not been finished yet. We hope to take a deep insight into the phenotypic characterization of transgenic *Arabidopsis* lines. It will be more profound if we get the relevant positive phenotypic results in the future.

## Data Availability Statement

The original contributions presented in the study are publicly available. This data can be found here: National Center for Biotechnology Information (NCBI) BioProject database under accession number PRJNA751249.

## Author Contributions

XW, YuL, ZH, YC, DH, YK, and ZW conducted the experiments. YoL and BL organized and supervised the overall project. XW, LY, and HJ performed the data analysis and wrote the manuscript. BL edited the manuscript. All authors contributed to the article and approved the submitted version.

## Conflict of Interest

The authors declare that the research was conducted in the absence of any commercial or financial relationships that could be construed as a potential conflict of interest.

## Publisher’s Note

All claims expressed in this article are solely those of the authors and do not necessarily represent those of their affiliated organizations, or those of the publisher, the editors and the reviewers. Any product that may be evaluated in this article, or claim that may be made by its manufacturer, is not guaranteed or endorsed by the publisher.

## Footnotes

1
https://www.peanutbase.org/peanut_genome

2
https://cran.r-project.org/web/packages/pheatmap/

## Supplementary Material

Supplementary Table 1 qRT-PCR primers in this study.

Supplementary Table 2 A list of metabolites detected in the samples.

Supplementary Table 3 The list of differential changed metabolites between the two peanut varieties under cold stress.

Supplementary Table 4 Classification of differential changed metabolites between two peanut cultivars under cold stress.

Supplementary Table 5 The accumulation pattern of metabolites during cold stress in peanut.

Supplementary Table 6 Statistical analyses and mapping results of RNA sequencing reads.

Supplementary Table 7 Differentially expressed genes between cold-tolerant and cold-susceptible cultivars.

Supplementary Table 8 A list of cold-responsive genes in SLH and ZH12 peanut.

Supplementary Table 9 The detail information of 3,620 cold-tolerance genes.

Supplementary Table 10 GO enrichment analysis cold-tolerance genes.

Supplementary Table 11 KEGG pathway enrichment analysis of cold tolerance genes.

Supplementary Table 12 A gene list of WGCNA modules.

Supplementary Table 13 The top 20 hub genes in red and tan modules.

Supplementary Table 14 The transcription levels of antioxidative genes and calcium signal genes in the two cultivars under cold stress.

Supplementary Table 15 The transcription levels of CBF genes in the two cultivars under cold stress.
