# Supplementary material for: Integrated Transcriptomics and Metabolomics Analysis Reveal Key Metabolism Pathways Contributing to Cold Tolerance in Peanut
Source: Front Plant Sci. 2021 Nov 24;12:752474. doi: 10.3389/fpls.2021.752474 (PMC8652294; doi:10.3389/fpls.2021.752474)
Supplement: Supplementary file 16 [file Data_Sheet_1.PDF]

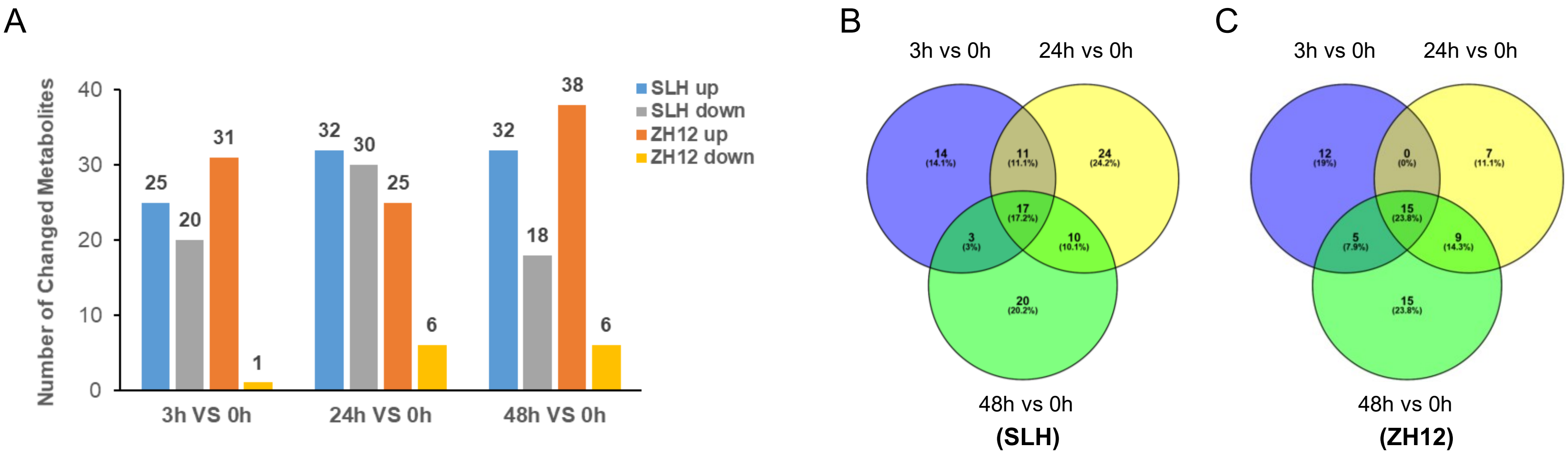

Figure S1. Summary of the significantly changed metabolites of peanut in response to cold stress. (A) Number of increased or decreased metabolites of the two genotypes (SLH and ZH12) under cold stress. (B), (C) the Venn diagram of changed metabolites between different time points in SLH and ZH12.

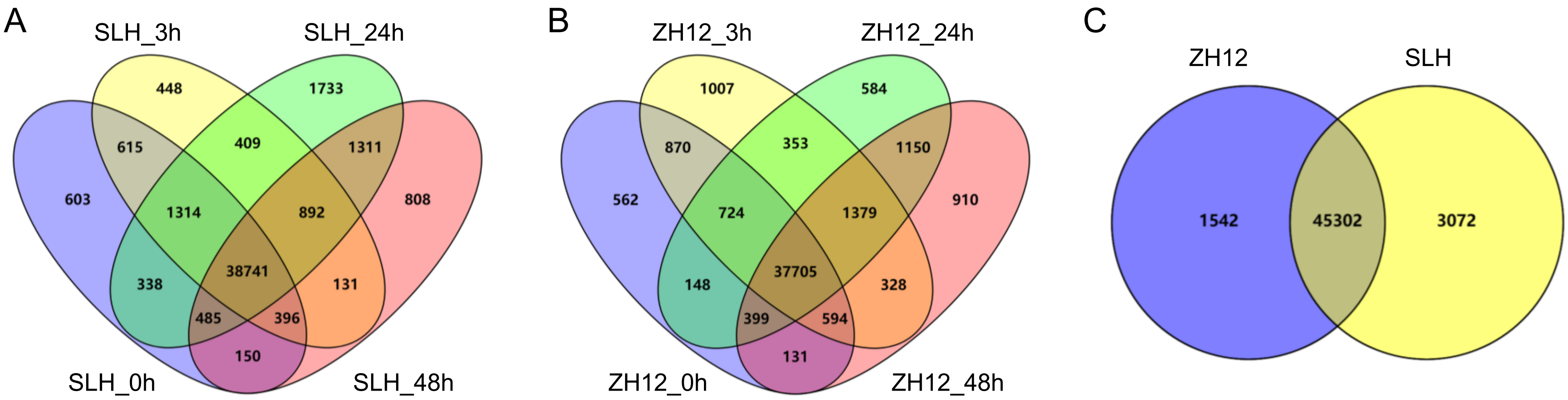

Figure S2. An overview of expressed genes in peanut. (A) (B), The shared and specifically expressed genes (FPKM > 0.1) in control (0h) and different cold treatment time points (3h, 24h and 48h) in SLH and ZH12, respectively. (C), The shared and uniquely of all the expressed gene (FPKM > 0.1) between two peanut genotypes.

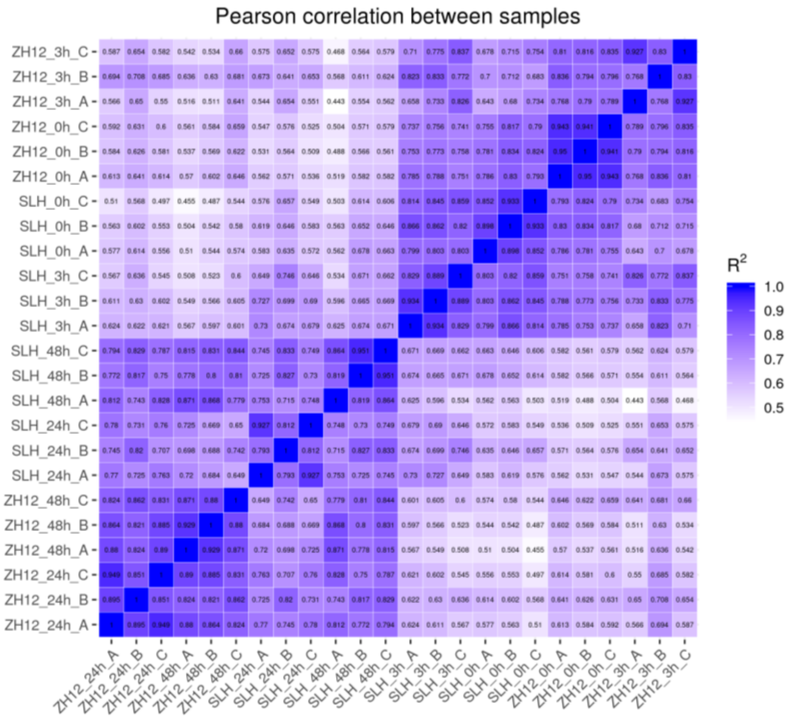

Figure S3. Pearson correlation analysis of transcriptome data between different samples. The Pearson correlation coefficients between different samples were calculated by the cor function in R package and presented as heatmap using pheatmap software.

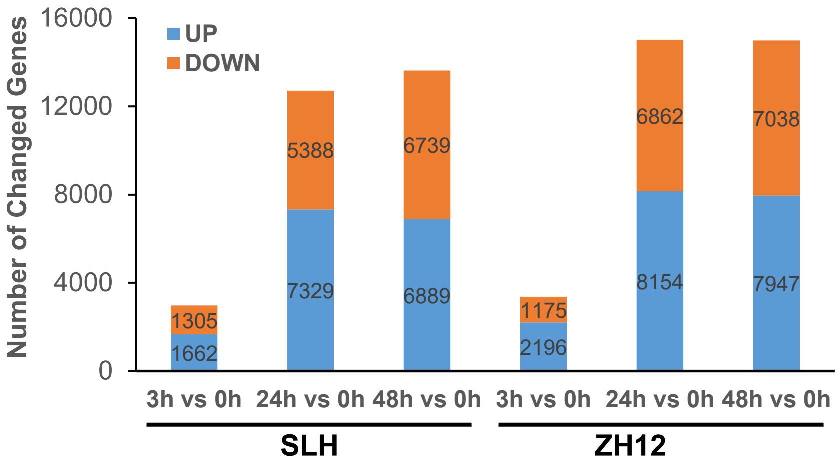

Figure S4. The number of differentially expressed genes (DEGs) in SLH and ZH12 exposed to cold treatment.

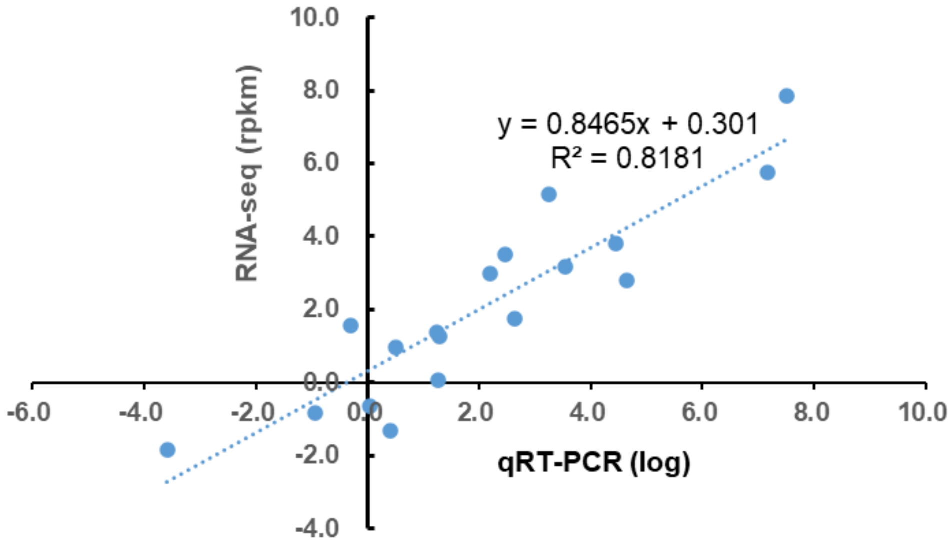

Figure S5. Correlation analysis of the RNA-seq data with the qRT-PCR results.

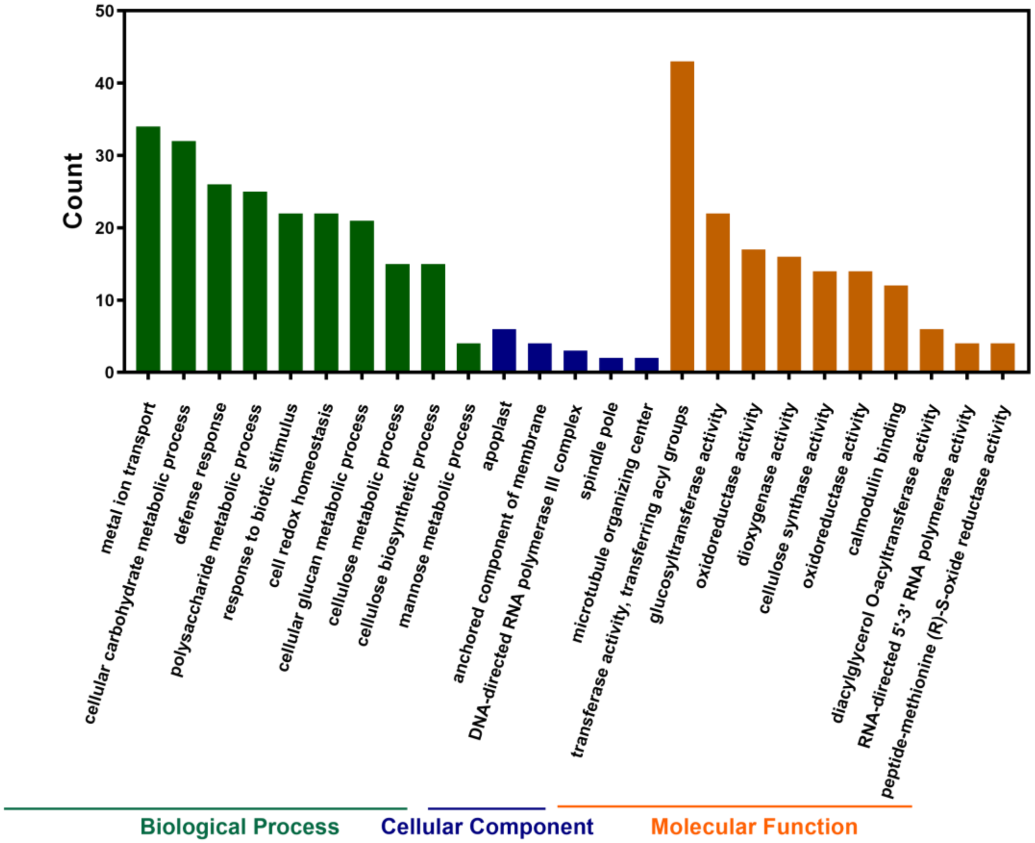

Figure S6. GO Functional enrichment analysis of 3620 cold tolerance genes in peanut.

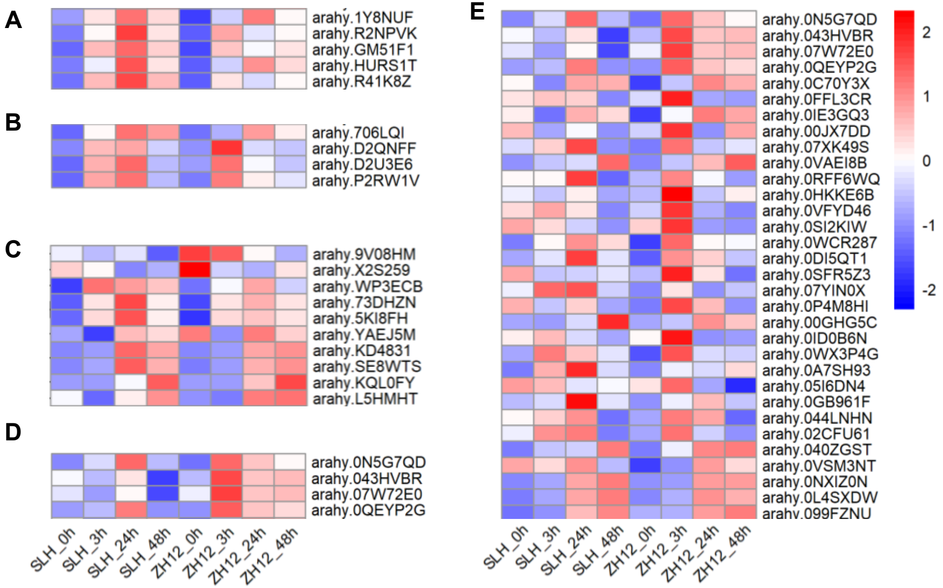

Figure S7. The transcriptional patterns of Calmodulin binding protein (A) and Calcium-dependent protein kinase (B), C-repeat Binding Factors (C) superoxide dismutase (D), Peroxidase (E), in response to cold stress. The heatmap values represent the Z-score of the FPKM (transcript levels) in different samples. Red color indicates high expression level, while blue is low.
